# Essays on Conceptual Electrochemistry: I. Bridging Open-Circuit Voltage of Electrochemical Cells and Charge Distribution at Electrode–Electrolyte Interfaces

**DOI:** 10.3389/fchem.2022.938064

**Published:** 2022-07-25

**Authors:** Jun Huang, Yufan Zhang

**Affiliations:** Institute of Energy and Climate Research, IEK-13, Theory and Computation of Energy Materials, Jülich, Germany

**Keywords:** open-circuit potential, electrochemical concept, electric double layer, surface charge, potential of zero charge

## Abstract

We ponder over how an electrochemical cell conforms itself to the open-circuit voltage (OCV) given by the Nernst equation, where properties of the electrodes play no role. We first show, *via* a pedagogical derivation of the Nernst equation, how electrode properties are canceled and then take a closer look into the electrode–electrolyte interface at one electrode by linking charge and potential distributions. We obtain an equilibrium Poisson–Nernst equation that shows how the charge distribution across an electrode–electrolyte interface can be dictated by the chemical potentials of redox species. Taking a ${\text{H}}_{2}/{\text{O}}_{2}$ fuel cell as an example, we demystify the formal analysis by showing how the two electrodes delicately regulate their “electron tails” to abide by the Nernst equation. In this example, we run into a seemingly counterintuitive phenomenon that two electrodes made of the same transition metal display two distinct potentials of zero charge. This example indicates that the double layer at transition metals with chemisorption can display distinct behaviors compared to ideally polarizable double layers at sp metals.

Many concepts in electrochemistry appear simple, but they are more complicated than they seem. One example is the electrode potential, which is among the first concepts we will encounter when opening any introductory electrochemistry textbook. However, the definition of absolute electrode potential had been extensively discussed in the 1970s–1980s (Trasatti, 1980; 1982; Trasatti, 1986a;b; Trasatti, 1986c; 1990). To illustrate, Trasatti (1990) stated that “the present author has considerably contributed to the discussion with several papers during the last 15 years in an attempt to bring the various views back to a unifying approach.” Trasatti’s formal theory states that “the absolute electrode potential is the difference in electronic energy between a point inside the metal (Fermi level) and a point outside the solution.” This definition has been adopted in the recommendation of The International Union of Pure and Applied Chemistry (IUPAC) (Trasatti, 1986a).

There is another “familiar stranger,” namely, the open-circuit voltage (OCV) of an electrochemical cell, which is simply the potential difference between two terminals of the cell under the open-circuit condition. Though the definition is unequivocal and the measurement easily accessible, the obtained value can be elusive and its interpretation disputed. A famous example is the OCV of a hydrogen/oxygen fuel cell at rest, also called the rest potential: why is the measured value significantly lower, by several hundreds of millivolts, than the thermodynamic value of 1.23 V *versus* the standard hydrogen electrode (SHE)?

James Hoare systematically investigated this problem in the 1960s–1970s (Hoare, 1962; Hoare, 1963; Hoare, 1964a; Hoare, 1964b; Hoare, 1964c; Thacker and Hoare, 1971; Hoare, 1974; Hoare, 1978). He concluded that the measured rest potential is a mixed potential of the oxygen reduction reaction and another parasitic reaction, namely, water dissociation reaction forming adsorbed hydroxyl. There are continued interests in understanding the rest potential of practical fuel cells beyond the glass cells used in Hoare’s experiments (Zhang et al., 2006; Vilekar and Datta, 2010; Reimer et al., 2019). In these practical situations, other factors such as hydrogen crossover come into play.

As an established fact, the OCV of the full cell calculated by the Nernst equation is independent of electrode properties, such as the chemical potential of electrons, the work function, and the valency electron density. For example, the OCV of a hydrogen/oxygen fuel cell under standard conditions is 1.23 V, regardless of the electrode materials. A seemingly naïve question might be asked:

How does the OCV of an electrochemical cell abide by the Nernst equation by canceling off all electrode-specific properties in an amazingly precise manner?

Though the answer to this question is self-evident, as thermodynamic quantities should be path-independent, a revisit of it from an alternative view is not meaningless. It may reveal something interesting. This question is discussed herein by first presenting a pedagogical derivation of the OCV of a general electrochemical cell. By decomposing the OCV into several parts, we show explicitly how electrode-specific properties cancel each other. Further delving into the electrode–electrolyte interface at one electrode, we link the potential difference between electrode and electrolyte phases with the net charge distribution at the electrode–electrolyte interface, which is then correlated with the chemical potentials of redox species involved in the reaction occurring on this electrode. The formal analysis is then demystified by taking an H_2_/O_2_ fuel cell as an example. In this example, we run into a weird point that two identical platinum electrodes constituting a hydrogen-oxygen fuel cell have different potentials of zero charge. We close this essay by commenting on why Trasatti’s relationship between the potential of the zero charge of an electrochemical interface and the work function of metals does not apply to the present case.

For an overall electrochemical reaction aA+bB=cC+dD , the Nernst equation for the cell relates the OCV under any condition ( E ) to that under standard conditions ( $E^{0}$ ) and the species activities ( $a_{\text{i}},\;\text{i}=\text{A},\text{ B},\text{ C},\text{ D}$ ), written as

$$E=E^{0}-\frac{RT}{nF}\ln\frac{a_{\text{C}}^{c}a_{\text{D}}^{d}}{a_{\text{A}}^{a}a_{\text{B}}^{b}}$$ (1)

with n being the number of electrons transferred, and $E^{0}$ is calculated by

$$E^{0}=-\frac{\text{Δ}G_{\text{r}}^{0}}{nF}$$ (2)

$\text{Δ}G_{\text{r}}^{0}$ is the Gibbs energy change under standard conditions, calculated by

$$\text{Δ}G_{\text{r}}^{0}=c\mu_{\text{C}}^{0}+d\mu_{\text{D}}^{0}-a\mu_{\text{A}}^{0}-b\mu_{\text{B}}^{0}$$ (3)

where $\mu_{i}^{\text{o}}$ ( i=A, B, C, D ) is the chemical potential of species i under standard conditions. Indeed, there is no electrode-specific property in the above equations for OCV.

Now, we will formulate the OCV in another way and decouple it into a serial connection of several potential differences. OCV is measured by connecting the cathode and anode with a voltmeter, as shown in Figure 1. The voltmeter has extremely high resistance, so a circuit connected with it allows for negligible current and thereby can be taken as an open circuit. The wires that connect the voltmeter with the two electrodes are of the same metal. The value on the voltmeter, herein the OCV, is expressed by the difference in the Fermi levels of these two wires:

$$E=\frac{\epsilon_{\text{F},\text{wc}}-\epsilon_{\text{F},\text{wa}}}{-e}\;$$ (4)

with $\epsilon_{\text{F},\text{wi}}=\mu_{\text{e},\text{wi}}-e\phi_{\text{wi}}$ and $\phi_{\text{wi}}$ (i = a, c) denote the respective inner potential of the metal wires at the cathode side and anode side. As, for the same material, $\mu_{\text{wc}}=\mu_{\text{wa}}$ , we arrive at the OCV:

$$E=\phi_{\text{wc}}-\phi_{\text{wa}}$$ (5)

**FIGURE 1:**
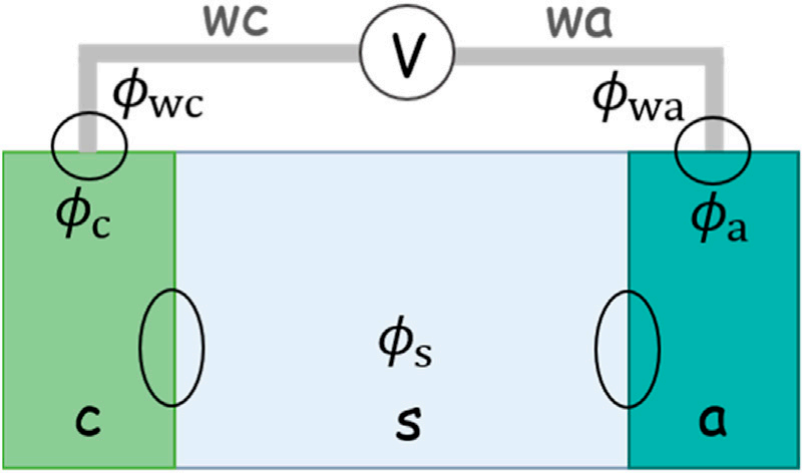
Schematic of an electrochemical cell. A voltmeter connects the cathode and anode to measure the open-circuit potential. The two wires of the voltmeter are made of the same metal.

The difference in the potential in two metal wires can be decoupled into a serial connection of four potential differences:

$$\phi_{\text{wc}}-\phi_{\text{wa}}={\text{Δ}}^{\text{wc}}\phi^{\text{c}}+{\text{Δ}}^{\text{c}}\phi^{\text{s}}+{\text{Δ}}^{\text{s}}\phi^{\text{a}}+{\text{Δ}}^{\text{a}}\phi^{\text{wa}}$$ (6)

with ${\text{Δ}}^{\text{x}}\phi^{\text{y}}=\phi^{\text{x}}-\phi^{y}$ . These four ${\text{Δ}}^{\text{x}}\phi^{\text{y}}$ in Eq. 6 correspond to the four circles in Figure 1. The first and the last terms are the metal contact potential difference, whereas the other two are the potential difference at the metal–solution interface. We will express them one by one.

The metal contact potential difference can be formulated with the condition that the electrons in connected metals are in electrochemical equilibrium (or Fermi levels align):

$$\mu_{\text{e},\text{wc}}-F\phi_{\text{wc}}=\mu_{\text{e},\text{c}}-F\phi_{\text{c}}$$ (7)

$$\mu_{\text{e},\text{wa}}-F\phi_{\text{wa}}=\mu_{\text{e},\text{a}}-F\phi_{\text{a}}$$ (8)

Combining Eqs 7, 8, the two chemical potentials in metal wires are canceled:

$${\text{Δ}}^{\text{wc}}\phi^{\text{c}}+{\text{Δ}}^{\text{a}}\phi^{\text{wa}}=\frac{\mu_{\text{e},\text{a}}}{F}-\frac{\mu_{\text{e},\text{c}}}{F}$$ (9)

We update Eq. 5 with the help of Eqs 6, 9:

$$E=\left({\text{Δ}}^{\text{c}}\phi^{\text{s}}-\frac{\mu_{\text{e},\text{c}}}{F}\right)-\left({\text{Δ}}^{\text{a}}\phi^{\text{s}}-\frac{\mu_{\text{e},\text{a}}}{F}\right)$$ (10)

where $\left({\text{Δ}}^{\text{i}}\phi^{\text{s}}-\frac{\mu_{\text{e},\text{i}}}{F}\right)$ (*i* = a, c) is one definition of absolute electrode potential (Trasatti, 1990). We are left with two potential differences at the metal–solution interface: ${\text{Δ}}^{\text{c}}\phi^{\text{s}}$ and ${\text{Δ}}^{\text{a}}\phi^{\text{s}}\text{a}$ .

Considering a redox couple in equilibrium on the cathode side, $\text{O}+n{\text{e}}^{-}=\text{R}$ . The charges bore in O and R are not explicitly expressed, but their sum must cancel the n electrons. Therefore, equilibrium gives

$$\mu_{\text{O},\text{c}}+nF\phi_{\text{s}}+n\mu_{\text{e},\text{c}}-nF\phi_{\text{c}}=\mu_{\text{R},\text{c}}$$ (11)

Some rearrangements give

$${\text{Δ}}^{\text{c}}\phi^{\text{s}}=\frac{\mu_{\text{O},\text{c}}-\mu_{\text{R},\text{c}}}{nF}+\frac{\mu_{\text{e},\text{c}}}{F}$$ (12)

In the same vein, ${\text{Δ}}^{\text{a}}\phi^{\text{s}}$ reads

$${\text{Δ}}^{\text{a}}\phi^{\text{s}}=\frac{\mu_{\text{O},\text{a}}-\mu_{\text{R},\text{a}}}{nF}+\frac{\mu_{\text{e},\text{a}}}{F}$$ (13)

Inserting Eqs 12, 13 back to Eq. 10, $\mu_{\text{e},\text{c}}$ and $\mu_{\text{e},\text{a}}$ are canceled:

$$E=\frac{\mu_{\text{O},\text{c}}-\mu_{\text{R},\text{c}}}{nF}-\frac{\mu_{\text{O},\text{a}}-\mu_{\text{R},\text{a}}}{nF}$$ (14)

Shown above is how all electrode-specific properties, namely, $\mu_{\text{e},\text{mc}}$ and $\mu_{\text{e},\text{ma}}$ , are canceled in the expression of OCV. If the chemical potential is expressed by the standard chemical potential and correction for the species activity is applied, Eq. 14 reduces back to Eq. 1.

Of note, a usual misunderstanding of the OCV reads $\text{OCV}={\text{Δ}}^{\text{c}}\phi^{\text{s}}-{\text{Δ}}^{\text{a}}\phi^{\text{s}}$ . Eq. 6 reminds us that the two contact potentials should never be forgotten. The correct equation for the OCV is Eq. 10.

Next, we look further into ${\text{Δ}}^{\text{i}}\phi^{\text{s}}\;\left(\text{i}=\text{a},\text{c}\right)$ . The distribution of electric potential from the electrode phase to the solution phase is governed by the Poisson equation:

∇.(ϵ(r)∇ϕ(r))=−ρ(r) (15)

where ϵ(r) is the dielectric permittivity, which is spatially inhomogeneous in the EDL and ρ(r) the net charge distribution, which can be decomposed into an electrode part $\rho_{\text{M}}\left(r\right)$ and a solution part $\rho_{\text{S}}\left(r\right)$ :

$$\rho \left(r\right)=\rho_{\text{M}}\left(r\right)+\rho_{\text{S}}\left(r\right)$$ (16)

We take some more lines to explain Eq. 16. This electrode–solution dichotomy of the net charge distribution is proper for the case without ion-specific adsorption. $\rho_{\text{M}}\left(r\right)$ is the sum of the negative charge carried by valence electrons of the electrode material and the positive charge of cationic cores of the electrode material. A small portion of valence electrons of the electrode will enter into the solution counterpart, termed the electron spillover phenomenon and called vividly “electron tail.” It can be modeled by the Thomas–Fermi theories (Badiali, 1987; Kornyshev, 1989; Schmickler, 1996; Huang, 2021; Huang et al., 2021). $\rho_{\text{S}}\left(r\right)$ is the sum of negative charge of anions and positive charge of cations in solution.

For the cases with ion-specific adsorption, chemical bonds are formed between the electrode and specifically adsorbed ions. In general, the specifically adsorbed ions are not electroneutral but still possess a fraction of charge (Schmickler and Guidelli, 2014). It then becomes principally difficult to separate $\rho_{\text{M}}\left(r\right)$ and $\rho_{\text{s}}\left(r\right)$ because ions, originally belonging to $\rho_{\text{s}}\;\left(r\right)$ , are now adsorbed onto the electrode with residual charge. A possible scheme is to treat the electrode together with the specifically adsorbed ions as a whole, whose net charge is denoted by $\rho_{\text{M}}\left(r\right)$ . This way, $\rho_{\text{s}}\left(r\right)$ is limited to the net charge of the nonspecifically absorbed ions in solution; see a recent Minireview on the surface charging behavior of EDLs with chemisorption (Huang, 2022).

For a one-dimensional case without ion-specific adsorption, where r is replaced with x , Eq. 15 reads

$$\frac{\text{d}\left(\epsilon \left(x\right)\frac{\text{d}\phi \left(x\right)}{\text{d}x}\right)}{\text{d}x}=-\rho \left(x\right)$$ (17)

In bulk solution, $\rho \left(x_{\text{s}}\right)=0$ and thus $\frac{\text{d}\phi \left(x_{\text{s}}\right)}{\text{d}x_{\text{s}}}=0$ . Integrating from $x_{\text{s}}$ to x gives

$$\epsilon \left(x\right)\frac{\text{d}\phi \left(x\right)}{\text{d}x}=\overset{x}{\underset{x_{\text{s}}}{\int }}-\rho \left(x\right)\text{d}x$$ (18)

Integrating again from $x_{\text{s}}$ to $x_{\text{i}}$ gives the potential difference across each electrode–electrolyte interface ( i=a,c ):

$${\text{Δ}}^{\text{i}}\phi^{\text{s}}=\overset{x_{\text{i}}}{\underset{x_{\text{s}}}{\int }}dx\frac{1}{\epsilon \left(x\right)}\overset{x}{\underset{x_{\text{s}}}{\int }}dx^{\prime }\rho_{\text{i}}\left(x^{\prime }\right)$$ (19)

with $x_{\text{s}}$ being the location of solution bulk and $x_{\text{i}}$ the location of the electrode bulk. Provided that dielectric properties of the electrode–solution interface are known *a priori*, ${\text{Δ}}^{\text{i}}\phi^{\text{s}}$ is exclusively determined by the net charge distribution $\rho_{\text{i}}\left(x\right)$ .

As no assumption on the condition of the interfacial reaction is made in the derivation, Eq. 19 is valid under both equilibrium and nonequilibrium conditions.

It has been established that the potential difference across the electrode–solution interface ${\text{Δ}}^{\text{i}}\phi^{\text{s}}$ is related to the chemical potentials of the redox species in solution *via* Eqs 12, 13. Now, with the help of Eq. 19, we arrive at an equilibrium Poisson–Nernst formula that bridges the net charge distribution at the electrode–electrolyte interface $\rho_{\text{i}}\left(x\right)$ with the chemical potentials:

$$\overset{x_{\text{i}}}{\underset{x_{\text{s}}}{\int }}dx\frac{1}{\epsilon \left(x\right)}\overset{x}{\underset{x_{\text{s}}}{\int }}dx^{\prime }\rho_{i}\left(x^{\prime }\right)=\frac{\mu_{\text{O},\text{i}}-\mu_{\text{R},\text{i}}}{nF}+\frac{\mu_{\text{e},\text{i}}}{F}$$ (20)

Eq. 20 implies that $\rho_{\text{i}}\left(x\right)$ can be tuned by the chemical potentials of redox species and also the electrons. Eq. 20 is valid under the equilibrium condition only due to the use of Eqs 12, 13.

Based on Eq. 20, two corollaries can be made: 1) for two electrodes of the same material where different redox species are in equilibrium, the net charge distribution will be different and 2) for two electrodes of different materials, even if the redox species—with whom they have established equilibrium—are the same, the net charge distribution will be different.

In order to understand the first corollary from a microscopic perspective, it is instructive to consider an H_2_/O_2_ fuel cell, schematically shown in Figure 2. Both electrodes made of platinum are immersed in an acidic solution with pH = 0. The cell is kept under the open-circuit condition.

**FIGURE 2:**
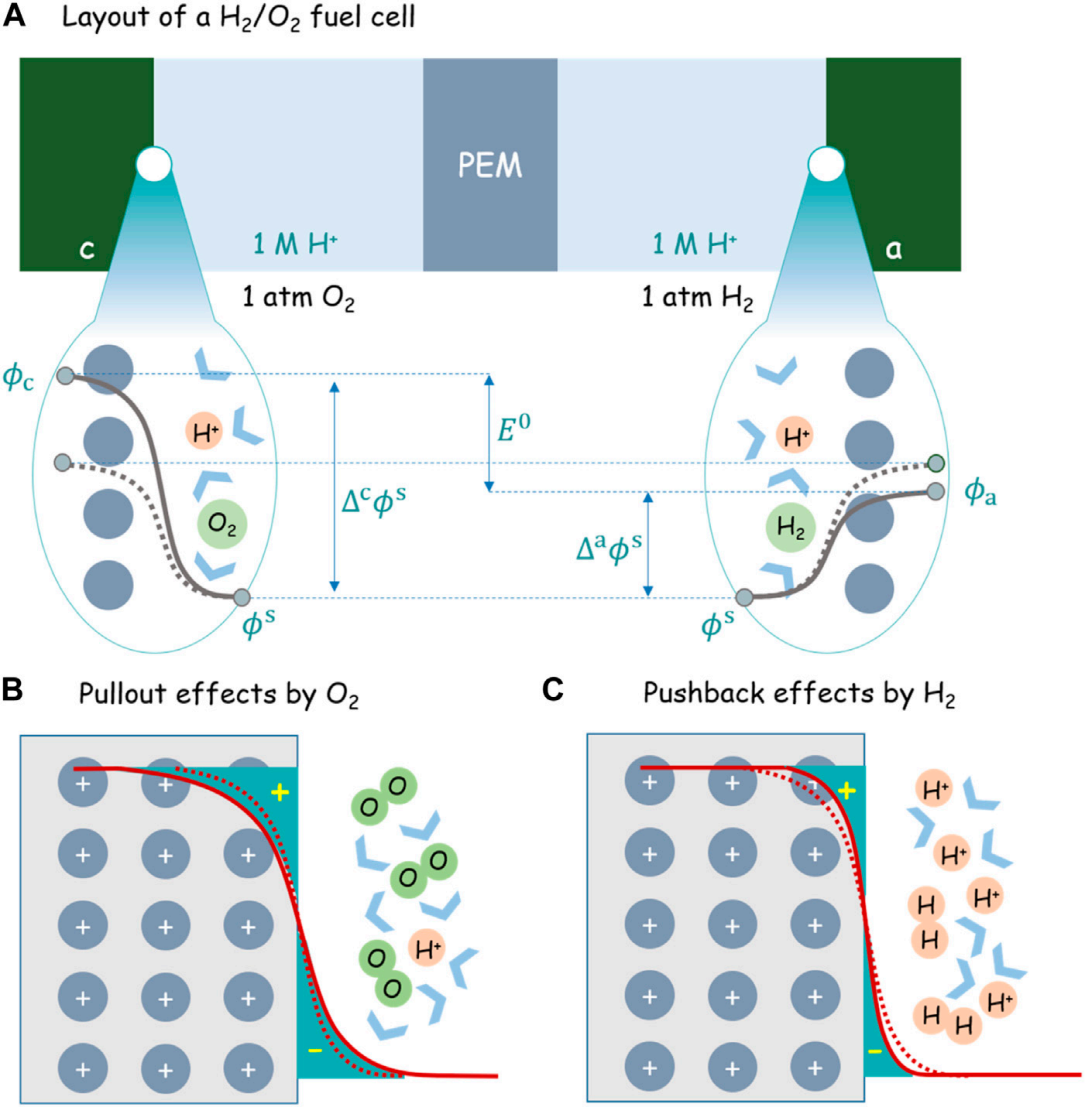
**(A)** Layout of an H_2_/O_2_ fuel cell under the standard condition (1 atm O_2_, 1 atm H_2_, 1M H^+^, and 25 °C). The solid and dashed gray lines denote, respectively, the potential distribution before and after H2 and O2 are injected into the cell. PEM is short for the proton exchange membrane. The schematically shown $E=\Delta^{c}\phi^{s}-\Delta^{a}\phi^{s}$ is only valid for the case of two electrodes made of identical material and thus $\mu_{e,c}=\mu_{e,a}$ . **(B)** and **(C)** show the pullout and pushback effects by oxygen and hydrogen on the metal electron density distribution, respectively. The dashed and solid red lines denote the electron density distribution before and after the injection of H_2_ and O_2_.

At the outset, no H_2_/O_2_ gas has been injected into the cell and dissolved in the solution. Therefore, the anode and cathode sides are totally symmetric in terms of electrode material and electrolyte component. Therefore, regardless of any reaction that may occur at both interfaces, we can safely state that ${\text{Δ}}^{\text{c}}\phi^{\text{s}}={\text{Δ}}^{\text{a}}\phi^{\text{s}}$ and the cell voltage is exactly zero, as schematically shown in Figure 2A.

Meanwhile, dashed lines in Figures 2B,C show schematically the electron density distribution at both electrodes for this symmetric case. The detailed charge distributions can be calculated using first-principles simulations based on the density-functional theory (DFT) (Le et al., 2017; Sakong and Groß, 2018, 2020; Le et al., 2021).

From now on, let oxygen and hydrogen gas come in the cathode and anode compartments while the external electric circuit is still kept open. The redox couple of $O_{2}$ , $H^{+}$ , and $H_{2}O$ occupies the cathode side, whereas that of $H^{+}$ and $H_{2}$ occupies the anode side. When the equilibrium for each redox couple is established respectively at the cathode and the anode, ${\text{Δ}}^{c}\phi^{s}$ and ${\text{Δ}}^{a}\phi^{s}$ are determined by Eqs 11, 12. Under standard conditions, the Nernst equation, Eq. 1, gives E=1.23 V . Any parasitic reaction that results in a mixed potential is not considered here.

Using Eq. 9 and recalling the two electrodes as both made of Pt, that is, $\mu_{\text{e},\text{c}}=\mu_{\text{e},\text{a}}$ , we have

$${\text{Δ}}^{\text{c}}\phi^{\text{s}}-{\text{Δ}}^{\text{a}}\phi^{\text{s}}=1.23\text{ V}$$ (21)

The differences between ${\text{Δ}}^{c}\phi^{s}$ and ${\text{Δ}}^{a}\phi^{s}$ must be ascribed to the difference in the net charge distribution ρ(x) , according to Eq. 19, which, in turn, is tuned by the chemical potentials of redox species, according to Eq. 20. As the electrochemical cell is kept under the ideal open-circuit condition, electrons cannot be exchanged between the two electrodes. On the cathode side, after oxygen gas is introduced, oxygen reduction reaction, O_2_ + 4H^+^ + 4e → 2H_2_O, must occur until it equilibrates with the reverse reaction. In other words, some electrons have been consumed in the cathode platinum to establish the equilibrium. Since the circuit is open and there is no way to compensate the electron consumption, the cathode platinum must be positively charged. Following the same line of reasoning, the anode platinum must be negatively charged due to excess electrons generated from hydrogen oxidation, H_2_ → 2H^+^ + 2e. However, the amount of excess on both electrodes is unknown without a model for the electrochemical double layers. Moreover, the presence of H_2_ and O_2_ also contribute to the change of net charge distribution, as shown in the dashed and solid curves in Figures 2B,C.

After introducing hydrogen and oxygen into the anode and cathode, hydrogen repels metal electrons back into the metal skeleton, while oxygen pulls more metal electrons out of the metal skeleton. The pullout effect by oxygen increases the surface dipole moment by increasing the distance between positively charged ionic cores and negatively charged “electron tails.” The pushback effect by the hydrogen, on the contrary, does the opposite. The uplift of ${\text{Δ}}^{\text{c}}\phi^{\text{s}}$ is a combined effect of positively charged electrode and the raised dipole moment due to the pullout effect. In the same logic, the suppression of ${\text{Δ}}^{\text{a}}\phi^{\text{s}}$ is a combined effect of negatively charged electrode and reduced dipole moment due to the pushback effect. The ultimate thrust for adjusting the free charge distribution is the thermodynamic requirement expressed in Eq. 20.

Theory and experiment revealed that the pushback effect of adsorbed hydrogen decreases the work function of Pt(111) (Li et al., 2021), whereas the pullout effect of adsorbed oxygen increases it (Malek and Eikerling, 2018). As regards the EDL at Pt(111) contacted with an acidic aqueous solution, a mean-field model has shown that chemisorption of partially charged hydroxyl and oxygen contributes an additional surface dipole moment, leading to a second pzc and an overall nonmonotonic surface charging behavior (Huang et al., 2016; Huang et al., 2018), which are confirmed in atomistic simulations (Fernandez-Alvarez and Eikerling, 2019; Tesch et al., 2021; Braunwarth et al., 2022).

Let us relook at this problem from the perspective of pzc. The pzc is defined as the potential at which no net charge is accumulated on the electrode. The anode herein is actually the standard hydrogen electrode (SHE). It has a potential of 0 V with reference to the SHE because it takes itself as the reference. Since the anode is negatively charged, it has a pzc higher than 0 V. The cathode has a potential of 1.23 V with reference to the SHE. Since it is positively charged, it has a pzc lower than 1.23 V. Due to the pullout and pushback effects, the two pzc are arguably not equal to each other.

Ostensibly, it is strange that the two electrodes, made of the same metal platinum, could have so different pzcs. The point is that the pzc is not a property of the electrode itself but a property of the electrode–electrolyte interface; the latter could be significantly changed by the electrolyte composition. That has been demonstrated in several experimental works (Smalley, 2017; Shatla et al., 2021) and discussed in a recent modeling work (Huang et al., 2020). As an immediate implication, it is ambiguous to say the pzc of an electrode material without specifying the adjacent electrolyte solution.

As a final remark, the present case where platinum can have two different pzcs contradicts Trasatti’s relationship between the pzc of an EDL and the work function of the metal constituting the EDL (Trasatti, 1971). It should be noted that Trasatti’s relationship was established for “clean” metal surfaces without adsorption or chemisorption. However, chemisorption occurs on the surfaces of two Pt electrodes for the present case where the surface structure of the metal changes from its original state. Therefore, our analysis indicates that Trasatti’s relationship does not apply to electrocatalytic EDLs, which display distinct behaviors compared to ideally polarizable EDLs at “clean” metal surfaces. For instance, no Gouy–Chapman minimum was observed in the differential double-layer capacitance curves of Pt(111), even in the so-called pure double-layer region (Pajkossy and Kolb, 2007; Ojha et al., 2020; Ojha et al., 2022). It is also important to notice that the usual pzc is measured under a closed-circuit condition, whereas the two platinum electrodes in our case are under open-circuit condition. Therefore, the pzc under the open-circuit condition could be different from that under the closed-circuit condition because the surface state of the electrodes changes.

In conclusion, we have touched upon the seemingly trivial question that how the electrochemical cell conforms itself to the OCV stipulated by the Nernst equation. We have obtained an equilibrium Poisson–Nernst equation in Eq. 20 relating the net charge distribution across the electrode–electrolyte interface to chemical potentials of redox species. Furthermore, an H_2_/O_2_ fuel cell has been used to illustrate how the OCV is generated microscopically *via* tuning the “electron tail,” namely, the spillover electron, in the EDL. It is important to note that the analysis has been limited to the equilibrium state under the open-circuit condition. The closed-circuit condition with charge transfer reactions occurring at both electrodes will be addressed in a separate essay.

## Data Availability

The original contributions presented in the study are included in the article/supplementary material. Further inquiries can be directed to the corresponding author.

## References

[B1] Badiali J. P. (1987). The Jellium Model in Electrochemistry. Berichte Bunsenges. für Phys. Chem. 91, 270–276. 10.1002/bbpc.19870910406

[B2] Braunwarth L. Jung C. Jacob T. (2022). Potential-dependent Pt (111)—water Interface: Tackling the Challenge of a Consistent Treatment of Electrochemical Interfaces. 10.26434/chemrxiv-2022-xndvx PMC10092414 36123306

[B3] Fernandez-Alvarez V. M. Eikerling M. H. (2019). Interface Properties of the Partially Oxidized Pt(111) Surface Using Hybrid DFT-Solvation Models. ACS Appl. Mat. Interfaces 11, 43774–43780. 10.1021/acsami.9b16326 31650835

[B4] Hoare J. P. (1963). A Study of the Rest Potentials in the Gold-Oxygen-Acid System. J. Electrochem. Soc. 110, 245. 10.1149/1.2425724

[B5] Hoare J. P. (1964b). On the Mixed Potentials Observed in the Iridium-Oxygen-Acid System. J. Electrochem. Soc. 111, 988. 10.1149/1.2426305

[B6] Hoare J. P. (1978). On the Normal Oxygen Potential at a Platinum‐Oxygen Alloy Diaphragm Electrode. J. Electrochem. Soc. 125, 1768–1771. 10.1149/1.2131291

[B7] Hoare J. P. (1962). Rest Potentials in the Platinum-Oxygen-Acid System. J. Electrochem. Soc. 109, 858. 10.1149/1.2425569

[B8] Hoare J. P. (1964c). Some Double-Layer Capacity Measurements on Platinum Electrodes. Nature 204, 71–73. 10.1038/204071b0 14240118

[B9] Hoare J. P. (1964a). The Effect of Metal Dissolution on the Rest Potential in the Palladium-Oxygen-Acid System. J. Electrochem. Soc. 111, 610. 10.1149/1.2426193

[B10] Hoare J. P. (1974). The Effect of Oxygen Dissolved in Pt on the Potential of a Pt∕O2 Electrode at Rest. J. Electrochem. Soc. 121, 872. 10.1149/1.2401940

[B11] Huang J. Chen S. Eikerling M. (2021). Grand-Canonical Model of Electrochemical Double Layers from a Hybrid Density–Potential Functional. J. Chem. Theory Comput. 10.1021/acs.jctc.1c00098 33787259

[B12] Huang J. (2021). Hybrid Density-Potential Functional Theory of Electric Double Layers. Electrochimica Acta 389, 138720. 10.1016/j.electacta.2021.138720

[B13] Huang J. Li P. Chen S. (2020). Potential of Zero Charge and Surface Charging Relation of Metal-Solution Interphases from a Constant-Potential Jellium-Poisson-Boltzmann Model. Phys. Rev. B 101, 125422. 10.1103/physrevb.101.125422

[B14] Huang J. Malek A. Zhang J. Eikerling M. H. (2016). Non-monotonic Surface Charging Behavior of Platinum: A Paradigm Change. J. Phys. Chem. C 120, 13587–13595. 10.1021/acs.jpcc.6b03930

[B15] Huang J. (2022). Surface Charging Behaviors of Electrocatalytic Interfaces with Partially Charged Chemisorbates. Curr. Opin. Electrochem. 33, 100938. 10.1016/j.coelec.2022.100938

[B16] Huang J. Zhou T. Zhang J. Eikerling M. (2018). Double Layer of Platinum Electrodes: Non-monotonic Surface Charging Phenomena and Negative Double Layer Capacitance. J. Chem. Phys. 148, 044704. 10.1063/1.5010999 29390839

[B17] Kornyshev A. A. (1989). Metal Electrons in the Double Layer Theory. Electrochimica Acta 34, 1829–1847. 10.1016/0013-4686(89)85070-4

[B18] Le J. Iannuzzi M. Cuesta A. Cheng J. (2017). Determining Potentials of Zero Charge of Metal Electrodes versus the Standard Hydrogen Electrode from Density-Functional-Theory-Based Molecular Dynamics. Phys. Rev. Lett. 119, 016801. 10.1103/PhysRevLett.119.016801 28731734

[B19] Le J. B. Fan Q. Y. Li J. Q. Cheng J. (2020). Molecular Origin of Negative Component of Helmholtz Capacitance at Electrified Pt (111)/Water Interface. Science Advances 6 (41), eabb1219. 33028519 10.1126/sciadv.abb1219 PMC7541063

[B20] Li P. Huang J. Hu Y. Chen S. (2021). Establishment of the Potential of Zero Charge of Metals in Aqueous Solutions: Different Faces of Water Revealed by Ab Initio Molecular Dynamics Simulations. J. Phys. Chem. C 125, 3972–3979. 10.1021/acs.jpcc.0c11089

[B21] Malek A. Eikerling M. H. (2018). Chemisorbed Oxygen at Pt(111): a DFT Study of Structural and Electronic Surface Properties. Electrocatalysis 9, 370–379. 10.1007/s12678-017-0436-0

[B22] Ojha K. Arulmozhi N. Aranzales D. Koper M. T. M. (2020). Double Layer at the Pt(111)-Aqueous Electrolyte Interface: Potential of Zero Charge and Anomalous Gouy-Chapman Screening. Angew. Chem. Int. Ed. 59, 711–715. 10.1002/anie.201911929 PMC6973170 31682314

[B23] Ojha K. Doblhoff-Dier K. Koper Marc T. M. (2022). Double-layer Structure of the Pt(111)–Aqueous Electrolyte Interface. Proc. Natl. Acad. Sci. 119, e2116016119. 10.1073/pnas.2116016119 35042778 PMC8784099

[B24] Pajkossy T. Kolb D. M. (2007). Double Layer Capacitance of the Platinum Group Metals in the Double Layer Region. Electrochem. Commun. 9, 1171–1174. 10.1016/j.elecom.2007.01.002

[B25] Reimer U. Cai Y. Li R. Froning D. Lehnert W. (2019). Time Dependence of the Open Circuit Potential of Platinum Disk Electrodes in Half Cell Experiments. J. Electrochem. Soc. 166, F3098–F3104. 10.1149/2.0121907jes

[B26] Sakong S. Groß A. (2018). The Electric Double Layer at Metal-Water Interfaces Revisited Based on a Charge Polarization Scheme. J. Chem. Phys. 149, 084705. 10.1063/1.5040056 30193475

[B27] Sakong S. Groß A. (2020). Water Structures on a Pt(111) Electrode from Ab Initio Molecular Dynamic Simulations for a Variety of Electrochemical Conditions. Phys. Chem. Chem. Phys. 22, 10431–10437. 10.1039/c9cp06584a 31976502

[B28] Schmickler W. (1996). Electronic Effects in the Electric Double Layer. Chem. Rev. 96, 3177–3200. 10.1021/cr940408c 11848857

[B29] Schmickler W. Guidelli R. (2014). The Partial Charge Transfer. Electrochimica Acta 127, 489–505. 10.1016/j.electacta.2014.02.057

[B30] Shatla A. S. Landstorfer M. Baltruschat H. (2021). On the Differential Capacitance and Potential of Zero Charge of Au(111) in Some Aprotic Solvents. ChemElectroChem 8, 1817–1835. 10.1002/celc.202100316

[B31] Smalley J. F. (2017). Potential of Zero Charge and its Temperature Derivative for Au(111) Electrode|Alkanethiol SAM|1.0 M Aqueous Electrolyte Solution Interfaces: Impact of Electrolyte Solution Ionic Strength and its Effect on the Structure of the Modified Electrode|Electrolyte Solution Interface. J. Phys. Chem. C 121, 9260–9272. 10.1021/acs.jpcc.6b10954

[B32] Tesch R. Kowalski P. M. Eikerling M. H. (2021). Properties of the Pt(111)/electrolyte Electrochemical Interface Studied with a Hybrid DFT-Solvation Approach. J. Phys. Condens. Matter 33, 444004. 10.1088/1361-648x/ac1aa2 34348250

[B33] Thacker R. Hoare J. P. (1971). Sorption of Oxygen from Solution by Noble Metals. J. Electroanal. Chem. Interfacial Electrochem. 30, 1–14. 10.1016/0368-1874(71)85027-x

[B34] Trasatti S. (1986b). Components of the Absolute Electrode Potential. Conceptions and Misinterpretations. Mater. Chem. Phys. 15, 427–438. 10.1016/0254-0584(86)90026-x

[B35] Trasatti S. (1986c). “Components of the Electrode Potential. Concepts and Problems,” in Trends in Interfacial Electrochemistry. Editor Silva A. F. (Dordrecht: Springer Netherlands), 1–24. 10.1007/978-94-009-4694-1_1

[B36] Trasatti S. (1990). The "absolute" Electrode Potential-The End of the Story. Electrochimica Acta 35, 269–271. 10.1016/0013-4686(90)85069-y

[B37] Trasatti S. (1986a). The Absolute Electrode Potential: an Explanatory Note (Recommendations 1986). Pure Appl. Chem. 58, 955–966. 10.1351/pac198658070955

[B38] Trasatti S. (1982). The Concept and Physical Meaning of Absolute Electrode Potential. J. Electroanal. Chem. Interfacial Electrochem. 139, 1–13. 10.1016/0022-0728(82)85100-0

[B39] Trasatti S. (1980). “The Electrode Potential,” in Comprehensive Treatise of Electrochemistry: The Double Layer. Editors Bockris J. O. M. Conway B. E. Yeager E. (Boston, MA: Springer US), 45–81. 10.1007/978-1-4615-6684-7_2

[B40] Trasatti S. (1971). Work Function, Electronegativity, and Electrochemical Behaviour of Metals. J. Electroanal. Chem. Interfacial Electrochem. 33, 351–378. 10.1016/s0022-0728(71)80123-7

[B41] Vilekar S. A. Datta R. (2010). The Effect of Hydrogen Crossover on Open-Circuit Voltage in Polymer Electrolyte Membrane Fuel Cells. J. Power Sources 195, 2241–2247. 10.1016/j.jpowsour.2009.10.023

[B42] Zhang J. Tang Y. Song C. Zhang J. Wang H. (2006). PEM Fuel Cell Open Circuit Voltage (OCV) in the Temperature Range of 23°C to 120°C. J. Power Sources 163, 532–537. 10.1016/j.jpowsour.2006.09.026

