# Corrigendum: Essays on conceptual electrochemistry: I. Bridging open-circuit voltage of electrochemical cells and charge distribution at electrode–electrolyte interfaces

**DOI:** 10.3389/fchem.2022.1095988

**Published:** 2022-11-30

**Authors:** Jun Huang, Yufan Zhang

**Affiliations:** Institute of Energy and Climate Research, IEK-13, Theory and Computation of Energy Materials, Jülich, Germany

**Keywords:** open-circuit potential, electrochemical concept, electric double layer, surface charge, potential of zero charge

In the original article, there was an error concerning the charge state of the two electrodes.

A correction has been made in the first paragraph after Eq. 21 on page 5. The text previously stated:

“Therefore, the total amount of free charge on both electrode surfaces remains zero. However, the distribution of the net charge is changed by the presence of H_2_ and O_2_, as shown in the dashed and solid curves in Figures 2B,C2.”

The corrected text appears below:

“On the cathode side, after oxygen gas is introduced, oxygen reduction reaction, $O_{2}+4H^{+}+4e\to 2H_{2}O$ , must occur until it equilibrates with the reverse reaction. In other words, some electrons have been consumed in the cathode platinum to establish the equilibrium. Since the circuit is open and there is no way to compensate the electron consumption, the cathode platinum must be positively charged. Following the same line of reasoning, the anode platinum must be negatively charged due to excess electrons generated from hydrogen oxidation, $H_{2}\to 2H^{+}+2e$ . However, the amount of excess charge on both electrodes is unknown without a model for the electrochemical double layers. Moreover, the presence of H_2_ and O_2_ also contribute to the change of net charge distribution, as shown in the dashed and solid curves in Figures 2B,C.”

A correction has been made to the second paragraph after Eq. 21 on page 5. The text previously stated:

“Therefore, ${∆}^{c}\phi^{s}$ increases. The pushback effect by the hydrogen, on the contrary, does the opposite. This is the microscopic origin of different ${∆}^{i}\phi^{s}$ at two sides.”

The corrected text appears below:

“The pushback effect by the hydrogen, on the contrary, does the opposite. The uplift of ${∆}^{c}\phi^{s}$ is a combined effect of positively charged electrode and the raised dipole moment due to the pullout effect. In the same logic, the suppression of ${∆}^{a}\phi^{s}$ is a combined effect of negatively charged electrode and reduced dipole moment due to the pushback effect.”

A correction has been made to the fourth paragraph after Eq. 21 on page 5. The text previously stated:

“The open-circuit condition exactly fulfills this condition. Therefore, ${∆}^{c}\phi^{s}$ and ${∆}^{a}\phi^{s}$ analyzed above can be discussed in the realm of pzc. The anode herein is actually the standard hydrogen electrode (SHE). It has a pzc of 0 V with reference to the SHE because it takes itself as the reference. As for the cathode, it has a pzc of 1.23 V with reference to the SHE due to Eq. 21.”

The corrected text appears below:

“The anode herein is actually the standard hydrogen electrode (SHE). It has a potential of 0 V with reference to the SHE because it takes itself as the reference. Since the anode is negatively charged, it has a pzc higher than 0 V. The cathode has a potential of 1.23 V with reference to the SHE. Since it is positively charged, it has a pzc lower than 1.23 V. Due to the pullout and pushback effects, the two pzc are arguably not equal to each other.”

A correction has been made to the sixth paragraph after Eq. 21 on page 5. The text previously stated:

“It should be noted that Trasatti’s relationship was established for sp metals without specific adsorption or chemisorption. However, chemisorption occurs on the surface of two Pt electrodes for the present case. Therefore, our analysis indicates that Trasatti’s relationship does not apply to electrocatalytic EDLs, which display distinct behaviors compared to ideally polarizable EDLs at sp metals.”

The corrected text appears below:

“It should be noted that Trasatti’s relationship was established for “clean” metal surfaces without adsorption or chemisorption. However, chemisorption occurs on the surfaces of two Pt electrodes for the present case where the surface structure of the metal changes from its original state. Therefore, our analysis indicates that Trasatti’s relationship does not apply to electrocatalytic EDLs, which display distinct behaviors compared to ideally polarizable EDLs at “clean” metal surfaces.”

The authors apologize for this error and state that this does not change the scientific conclusions of the article in any way. The original article has been updated.

